# Interleukin-15 Dendritic Cells Harness NK Cell Cytotoxic Effector Function in a Contact- and IL-15-Dependent Manner

**DOI:** 10.1371/journal.pone.0123340

**Published:** 2015-05-07

**Authors:** Sébastien Anguille, Heleen H. Van Acker, Johan Van den Bergh, Yannick Willemen, Herman Goossens, Viggo F. Van Tendeloo, Evelien L. Smits, Zwi N. Berneman, Eva Lion

**Affiliations:** 1 Laboratory of Experimental Hematology, Tumor Immunology Group (TIGR), Vaccine & Infectious Disease Institute (VAXINFECTIO), University of Antwerp, Faculty of Medicine and Health Sciences, Antwerp, Belgium; 2 Center for Cell Therapy & Regenerative Medicine, Antwerp University Hospital, Edegem, Belgium; 3 Center for Oncological Research, University of Antwerp, Faculty of Medicine and Health Sciences, Antwerp, Belgium; Baylor College of Medicine, UNITED STATES

## Abstract

The contribution of natural killer (NK) cells to the treatment efficacy of dendritic cell (DC)-based cancer vaccines is being increasingly recognized. Much current efforts to optimize this form of immunotherapy are therefore geared towards harnessing the NK cell-stimulatory ability of DCs. In this study, we investigated whether generation of human monocyte-derived DCs with interleukin (IL)-15 followed by activation with a Toll-like receptor stimulus endows these DCs, commonly referred to as “IL-15 DCs”, with the capacity to stimulate NK cells. In a head-to-head comparison with “IL-4 DCs” used routinely for clinical studies, IL-15 DCs were found to induce a more activated, cytotoxic effector phenotype in NK cells, in particular in the CD56^bright^ NK cell subset. With the exception of GM-CSF, no significant enhancement of cytokine/chemokine secretion was observed following co-culture of NK cells with IL-15 DCs. IL-15 DCs, but not IL-4 DCs, promoted NK cell tumoricidal activity towards both NK-sensitive and NK-resistant targets. This effect was found to require cell-to-cell contact and to be mediated by DC surface-bound IL-15. This study shows that DCs can express a membrane-bound form of IL-15 through which they enhance NK cell cytotoxic function. The observed lack of membrane-bound IL-15 on “gold-standard” IL-4 DCs and their consequent inability to effectively promote NK cell cytotoxicity may have important implications for the future design of DC-based cancer vaccine studies.

## Introduction

The recent licensing of several high-profile cancer immunotherapy products, such as the dendritic cell (DC)-based prostate cancer vaccine sipuleucel-T, has further cemented immunotherapy as the fourth pillar in cancer treatment alongside the three traditional treatment options (i.e. surgery, chemotherapy and radiation therapy) [1]. The term “cancer immunotherapy” covers a myriad of therapeutic approaches that are based on the long-standing knowledge that our immune system is capable of mounting a defense against tumor cells [1]. One of these approaches entails the use of DCs as cellular tools for anti-cancer immunization [2,3]. DC vaccine strategies seek to exploit the potent antigen-presenting properties of DCs to induce tumor antigen-specific cytotoxic T lymphocytes (CTLs) [2]. Such CTLs are capable of specifically recognizing target antigens bound to major histocompatibility complex (MHC) class I molecules on the tumor cell surface and of mediating subsequent tumor cell lysis [2].

With the principal mode of action being antigen presentation and stimulation of tumor antigen-specific CTL immunity, immunomonitoring of DC cancer vaccine studies has been predominantly centered on the adaptive arm of the immune system [4]. Hitherto, only little focus has been placed on the innate immune arm, in particular on the effects of DC vaccination on innate anti-tumor effectors such as natural killer (NK) cells [4–6]. Human NK cells are usually divided into two subsets based on their surface level of expression of CD56: CD56^bright^ and CD56^dim^ NK cells [7,8]. CD56^dim^ NK cells represent the predominant subset in the peripheral blood, constituting about 90% of the entire circulating NK cell population, whereas the reverse situation occurs in the lymph nodes [9]. Both subsets serve different, but not mutually exclusive, functions; CD56^bright^ NK cells are primarily responsible for cytokine production, such as IFN-γ, whereas CD56^dim^ NK cells are classically described as the more cytotoxic subset [7,8]. Among the key surface molecules involved in NK cell cytotoxic function are NKG2D, an activating immunoreceptor that plays an important role in target cell recognition, and natural cytotoxicity receptors (NCRs) such as NKp30 and NKp46 [10]. Unlike CTLs, NK cells mediate lysis of their target cells, including tumor cells, in a non-antigen-specific, non-MHC-restricted fashion [10]. This complementary anti-tumor activity places NK cells in a key position in the immune defense against cancer [10]. Less well acknowledged is that NK cells are also important for effective DC-based anti-tumor immunotherapy [4,5]. Nevertheless, a growing body of research indicates that the therapeutic activity of DC cancer vaccination does not exclusively rely on engagement of the adaptive immune arm, but also on its ability to harness innate anti-tumor effector cells [4,5]. The importance of NK cells in the anti-tumor activity of DC-based immunotherapy has been demonstrated in several animal studies, some of which have pointed to an indispensable role for NK cells in this respect [11–18]. In human clinical trials [19–21], the induction of NK cell immune responses by DC vaccination was shown to be associated with favorable clinical outcome, whereas such correlation has not been consistently reported for DC vaccine-induced T-cell immunity. Collectively, these data indicate that NK cells may be critical to successful anti-cancer immunization by DCs and implicate that, for optimal effectiveness, DC-based cancer vaccines should also possess NK cell-stimulatory activity [4,5].

Most clinical trials of DC-based cancer immunotherapy have been conducted using the so-called “interleukin (IL)-4 DC” type [3]. These IL-4 DCs are generated by *in vitro* culture of peripheral blood monocytes in the presence of granulocyte macrophage colony-stimulating factor (GM-CSF) and IL-4 for approximately 7 days. A mixture of the pro-inflammatory cytokines tumor necrosis factor (TNF)-α, IL-1β, IL-6 and prostaglandin E_2_ (PGE_2_) is usually added to the DC cultures 36–48 hr prior to the termination of the culture process for the purpose of inducing DC activation [3]. Although clinical benefit has been documented using this IL-4 DC-based approach, the past decade has witnessed the introduction of several new protocols that enable the generation of DCs with optimized anti-tumor immunostimulatory activity [3]. Within this context, we and others have reported a novel, rapid DC culture system in which highly immunostimulatory DCs are generated through monocyte culture in the presence of GM-CSF and IL-15, followed by activation/maturation of the resultant “IL-15 DCs” via Toll-like receptor (TLR) stimulation [22]. Such IL-15 DCs have already demonstrated their superiority over conventional IL-4 DCs in terms of their capacity to stimulate T helper type 1 (T_H_1)-, T helper type 17 (T_H_17)-, and CTL-immunity [23–29]. In addition, we have recently shown that IL-15 DCs possess direct tumoricidal activity [28,30], which may complement their T-cell stimulatory function [31,32].

Whether and how IL-15 DCs harness the anti-tumor potential of NK cells remains to be examined. Therefore, in this study, phenotypic and functional investigations were undertaken to determine and compare the NK cell-stimulatory capacity of IL-15 DCs and “gold-standard” IL-4 DCs, and to unravel the potential mechanisms involved therein.

## Materials and Methods

### Ethics statement

Peripheral blood mononuclear cells (PBMCs) were isolated by Ficoll density gradient centrifugation (Ficoll-Paque PLUS; GE Healthcare, Diegem, Belgium) from adult volunteer whole blood donations (supplied by the blood bank of the Red Cross Antwerp Blood Transfusion Center, Edegem, Belgium and by the Department of Hematology of the Antwerp University Hospital, Edegem, Belgium). Blood donor selection and collection was performed according to Belgian law and Belgian Red Cross policy, which includes the signing of a declaration in which the donor agrees that his/her blood donation can be used for purposes other than blood transfusion, including experimental research. The use of the donated blood for the specific experiments of this study was approved by the local Ethics Committee of the University of Antwerp (Antwerp, Belgium) under the reference number 11/47/366.

### Human cell lines

The human NK-sensitive K562 tumor cell line was obtained from the American Type Culture Collection (ATCC, Rockville, MD, USA; catalogue number: K-562 ATCC CCL243); the NK-resistant Daudi tumor cell line was obtained from ATCC (catalogue number: Daudi ATCC CCL-213) and provided to us by the laboratory of Prof K. Thielemans (Free University of Brussels, Brussels, Belgium). Both cell lines were maintained in Iscove’s modified Dulbecco’s medium (IMDM; Invitrogen, Merelbeke Belgium) supplemented with 10% fetal bovine serum (FBS; Invitrogen).

### DC culture

Immediately after isolation, PBMCs were further subfractioned into CD14^+^ monocytes and peripheral blood lymphocytes (PBLs) using a positive immunomagnetic cell selection kit (Miltenyi, Amsterdam, The Netherlands). IL-15 DCs were prepared from the CD14^+^ monocyte fraction as per our previously reported rapid DC culture protocol [22,25,28]. Monocyte differentiation into IL-15 DCs was induced with GM-CSF (800 IU/mL; Invitrogen) and IL-15 (200 ng/mL; Immunotools, Friesoythe, Germany). After 24–48 hr of differentiation, DCs were exposed for 16–20 hr to a DC maturation cocktail composed of the TLR7/8 ligand R-848 (3 μg/mL; Enzo Life Sciences, Antwerp, Belgium) in combination with TNF-α (2.5 ng/mL; Invitrogen), interferon (IFN)-γ (5000 IU/mL; Immunotools) and PGE_2_ (1 μg/mL; Pfizer, Puurs, Belgium). Control 7-day IL-4 DCs from the same blood donors were prepared and matured as previously described in detail [25]. All DC cultures were performed in 6-well culture plates (Corning Life Sciences, Schiphol-Rijk, The Netherlands; initial monocyte plating density of 1–1.2×10^6^ cells/mL) and maintained in Roswell Park Memorial Institute medium (RPMI-1640; Invitrogen) supplemented with 2.5% heat-inactivated human AB serum (hAB; Invitrogen).

### NK cell purification

Immediately after CD14^+^ immunomagnetic fractionation of the PBMCs (see above), the monocyte-depleted PBL fraction was subjected to a negative immunomagnetic selection procedure for isolation of untouched NK cells, according to the manufacturer’s protocol (Miltenyi). After purification, NK cells were frozen in 90% FBS supplemented with 10% dimethyl sulfoxide (DMSO; Sigma-Aldrich, Bornem, Belgium) and kept at -80°C until further use. Pilot experiments were carried out beforehand to determine whether the process of cryopreservation had any influence on NK cell functionality. These experiments confirmed that NK cells isolated and cryopreserved according to the above-described methodology fully retain their cytotoxic activity (data not shown). Based on these data and our previous observation that the cytokine-secretory capacity of NK cells is still maintained after cryopreservation [33], we proceeded to conduct all further experiments using purified cryopreserved NK cells.

### Flow cytometric immunophenotyping

Mature DCs were harvested, washed at least twice to remove any remnants of the DC maturation cocktail, and resuspended in fresh IMDM/10% FBS medium. DCs were then co-incubated with thawed autologous NK cells in 96-well culture plates (Corning) at a cell-to-cell ratio of 1:1 (10^5^ cells of each cell type) in a volume of 200 μL IMDM/10% FBS per well. After 24 hr and 48 hr of co-culture, NK cell immunophenotyping was performed using the following monoclonal antibodies (mAbs) and their corresponding isotype control mAbs: CD56-fluorescein isothiocyanate (FITC), CD69-allophycocyanin:cyanine 7 (APC:Cy7), CD25-phycoerythrin (PE), HLA-DR-allophycocyanin (APC), NKG2D-PE, NKp30-alexa fluor 647 (AF647) and NKp46-APC (all from Becton Dickinson [BD], Erembodegem, Belgium). Data were acquired on a FACSAria II flow cytometer (BD) and analyzed using FlowJo software (v10; Treestar, Ashland, OR, USA). After primary gating on the lymphocyte gate in forward scatter (FSC):side scatter (SSC) plots and subsequent exclusion of dead cells by 7-amino actinomycin D (7-AAD; BD) staining, mean fluorescence intensity (MFI) levels of each of the above surface markers were determined for the total CD56^+^ NK cell population as well as for the CD56^bright^ and CD56^dim^ subpopulations. The proportion (%) of NK cells positive for a given surface marker was calculated using the Overton histogram subtraction tool in FlowJo (subtracting the unstimulated NK cell control histogram from the DC-stimulated NK cell sample histogram).

For evaluation of membrane IL-15 expression, DC cultures were collected at different time points (monocyte stage, mature DC stage, 48 hr post-maturation, within the first hr after initiation of co-culture with NK cells and after 48 hr of co-culture with NK cells), incubated for 10 min with mouse gammaglobulins (Jackson ImmunoResearch, Suffolk, UK) to reduce non-specific Fcγ receptor binding, and then stained with IL-15-PE or isotype control mAb (R&D systems, Minneapolis, USA). Sample acquisition was performed on a FACScan (BD) or CyFlow ML (Partec, Münster, Germany) flow cytometer. Data were analyzed using FlowJo software and presented as histogram overlays.

### Cytokine secretion measurements

NK-DC co-cultures were set up as described above. After 48 hr, cell-free supernatants were harvested and stored at -20°C before analysis. Samples were assessed by using enzyme-linked immunosorbent assay (ELISA) for the presence of IFN-γ (Peprotech, Rocky Hill, NJ, USA) and by using electrochemiluminescence immunoassay (ECLIA; Meso Scale Discovery [MSD], Rockville, MD, USA) for the presence of GM-CSF, IFN-γ, IL-5, IL-8, IL-10, IL-13, MIP-1β, RANTES and TNF-α. Both assays were performed according to the manufacturer’s instructions. NK and DC monocultures were included as controls and used to calculate the expected cytokine concentrations in DC-NK co-cultures. Cytokine measurements were also performed on supernatants of DC-NK cell co-cultures stimulated for an additional 4 hr with the K562 cell line.

### Cytotoxicity assay and blocking experiments

Thawed NK cells and autologous mature DCs were co-cultured in triplicate in 96-well culture plates (Corning; 10^5^ cells of each cell type per well in a final volume of 300 μL IMDM/10% FBS). To examine the role of cell contact, analogous co-cultures were set up, except that the DCs were separated from the NK cells by a Transwell insert (Corning, 0.4 μm pore size). In some experiments, neutralizing anti-IL-15 mAb (10 μg/mL R&D; clone 34559) or corresponding mouse IgG_1_ isotype control mAb (R&D) was used in order to study the involvement of DC surface-bound IL-15. To ensure adequate blocking of IL-15, DCs were incubated for at least 1 hr with the above mAbs before starting co-culture with NK cells.

Target cells (K562 and Daudi) were labeled with PKH67 green fluorescent cell linker (Sigma-Aldrich), according to our previously reported technique [34], added to the NK cell +/- DC (co-)cultures for the last 4 hr of the culture at a single NK cell:target cell ratio of 5:1. Prior to flow cytometric acquisition (FACSAria II), triplicates were pooled and cell surface was stained with CD11c-V450 (BD) to discriminate target cells (PKH67^+^CD11c^-^) from NK cells (PKH67^-^CD11c^-^) and DCs (PKH67^-^CD11c^+^). Target cell death was determined by combined Annexin-V-APC (BD) / propidium iodide (PI; Sigma-Aldrich) staining, as detailed previously [28,34]. Cytotoxicity was calculated using the following formula [34]: % killing = 100 −[(% Annexin-V^-^/PI^-^ target cells with NK cells / % Annexin-V^-^/PI^-^ target cells without NK cells) x 100].

### Statistical analysis

GraphPad Prism software (v5.0; San Diego, CA, USA) was used for statistical analysis and graphing. Statistical comparisons were performed using repeated measures analysis of variance (ANOVA) with Bonferroni’s posthoc testing or, when indicated, using Wilcoxon matched pairs test. *P*-values <0.05 were considered statistically significant. All data were expressed as means ± standard error of the mean (SEM), unless specified otherwise.

## Results

### NK cells, in particular CD56^bright^ NK cells, express an activated cytotoxic effector phenotype upon IL-15 DC stimulation

**Fig 1:**
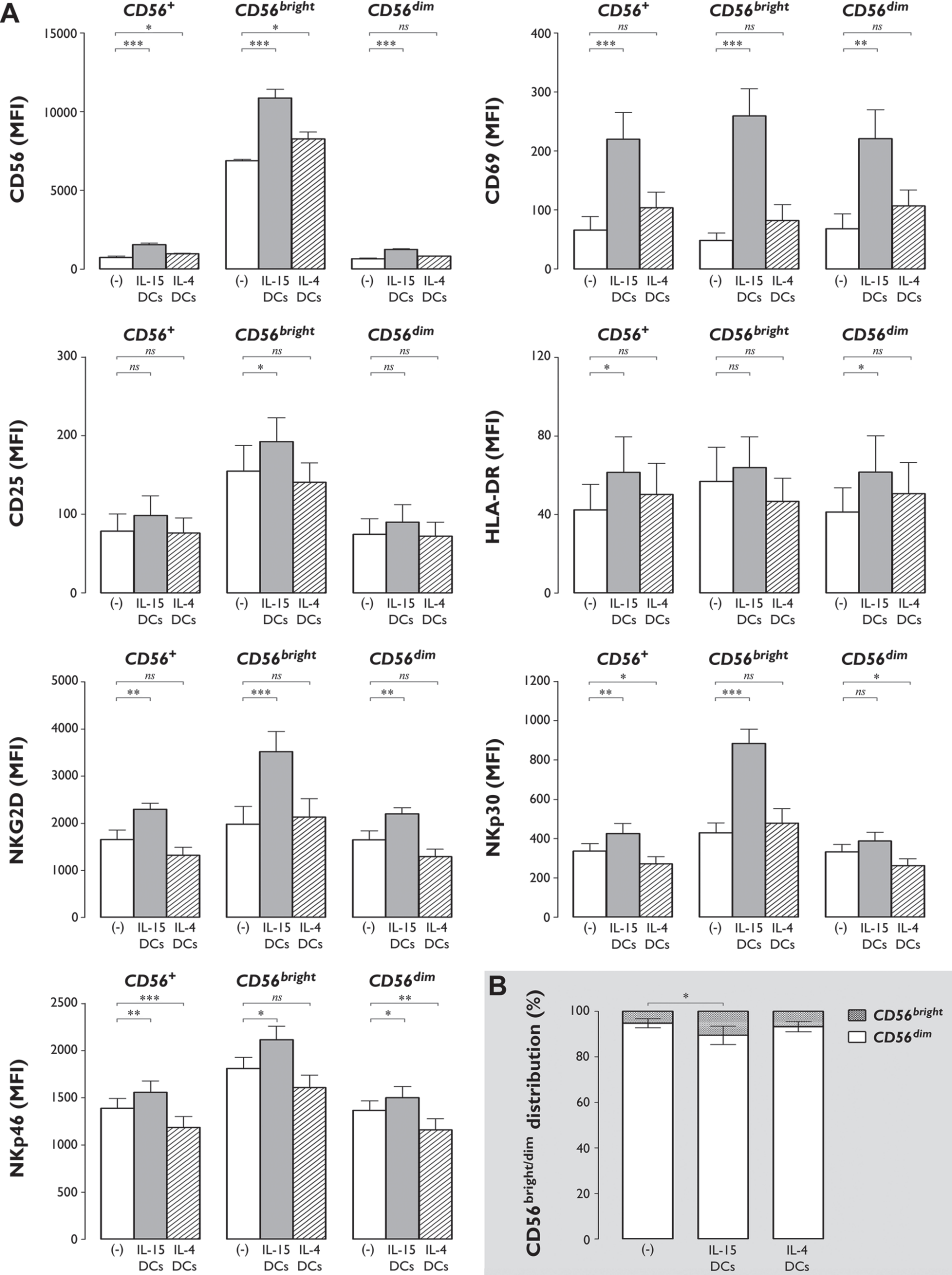
IL-15 DCs induce an activated cytotoxic effector phenotype in NK cells and enlarge the proportion of the CD56^bright^ NK cell subset. (**A**) Bar graphs showing the MFI (± SEM) of CD56, CD69, CD25, HLA-DR, NKG2D, NKp30 and NKp46 on unstimulated NK cells ((-), white bars) and NK cells stimulated for 48 hr with autologous IL-15 DCs (grey bars) or IL-4 DCs (dashed bars). Expression levels of the indicated markers are shown for the total CD56^+^ NK cell population, as well as for the CD56^bright^ and CD56^dim^ NK cell subpopulations. Data are from one experiment with 6 different donors (*, *P*<0.05; **, *P*< 0.01; ***, *P*<0.001; ns, not significant). (**B**) Bar graphs showing the relative distribution of CD56^bright^ and CD56^dim^ subsets in unstimulated NK cells ((-)) and in NK cells cultured for 48 hr with IL-15 DCs or IL-4 DCs. Data are expressed as mean (± SEM) percentages of 6 donors from 1 experiment (*, *P*<0.05).

Phenotypic characterization revealed a marked increase in expression level of the different NK cell surface antigens studied (CD56, CD69, CD25, HLA-DR, NKG2D, NKp30 and NKp46) following 48-hr co-culture of NK cells with autologous IL-15 DCs, in contrast to IL-4 DCs which possessed very limited capacity to induce NK cell activation (Fig 1 and Table 1). Most of the activation effects were already detected after 24 hr incubation (S1 Fig and S1 Table), but became even more distinct after 48 hr (Fig 1 and Table 1).

**Table 1:** Percentage increase in cell surface marker positivity compared with baseline after 48 hr stimulation of NK cells with either IL-15 DCs or IL-4 DCs.

	CD56^+^ NK cells		CD56^bright^ NK cells		CD56^dim^ NK cells	
	*IL-15 DCs*	*IL-4 DCs*	*IL-15 DCs*	*IL-4 DCs*	*IL-15 DCs*	*IL-4 DCs*
**CD56**	42.8±9.9%	20.1±9.5%*	51.7±14.3%	26.2±14.9%*	42.2±12.3%	19.8±10.3%*
**CD69**	33.6±11.1%	13.4±12.3%*	47.6±15.4%	15.3±7.8%*	32.6±11.0%	13.6±12.8%*
**CD25**	11.0±5.2%	4.5±3.8%*	16.8±10.5%	5.3±5.5%*	9.8±5.4%	4.3±3.6%*
**HLA-DR**	7.9±3.1%	3.7±1.3%*	7.9±6.0%	2.3±2.7%*	7.9±3.0%	3.9±1.4%*
**NKG2D**	25.7±15.2%	2.0±5.0%*	38.2±14.2%	11.4±8.6%*	24.1±14.4%	1.8±4.5%*
**NKp30**	13.3±8.4%	0.5±0.3%*	42.0±12.4%	10.5±9.5%*	9.8±8.5%	0.3±0.2%*
**NKp46**	11.1±6.3%	0.1±0.2%*	16.2±12.1%	3.1±5.4%*	10.3±6.4%	0.1±0.2%*

Pairwise comparison of the expression of CD56, CD69, CD25, HLA-DR, NKG2D, NKp30 and NKp46 on NK cells (total CD56^+^ NK cell population, CD56^bright^ subset and CD56^dim^ subset) after 48-hr incubation with IL-15 DCs or IL-4 DCs. Results are presented as mean (± SD) percentage increase in positivity of the indicated cell surface marker as compared with the baseline expression on unstimulated NK cells (calculated using the Overton histogram subtraction algorithm by subtracting the unstimulated NK cell control histogram from the DC-stimulated NK cell sample histogram). Asterisks indicate a statistically significant difference in cell surface marker expression between IL 15 DC-stimulated and IL-4 DC-stimulated NK cells (n = 6; *, *P*<0.05).

As shown in Fig 1A, stimulation of NK cells with IL-15 DCs significantly upregulated expression of CD56, an effect that was observed on both CD56^bright^ and CD56^dim^ subpopulations (*P*<0.001). IL-4 DCs induced a much less pronounced upregulation of CD56, which could be explained by the fact that only the smaller CD56^bright^ NK cell fraction displayed a significant increase in CD56 expression level (Fig 1A; *P*<0.05). Further subset analysis revealed that IL-15 DCs, but not IL-4 DCs, induced a marked increase in the CD56^bright^/CD56^dim^ NK cell ratio (Fig 1B; % CD56^bright^ NK cells in IL-15 DC-stimulated NK cell cultures *vs*. unstimulated NK cells: 10.6±4.0% *vs*. 5.2±2.0%, *P*<0.05).

NK cells displayed no significant alterations in expression level of the activation markers CD69, CD25 and HLA-DR upon exposure to IL-4 DCs (Fig 1A). Stimulation with IL-15 DCs caused a profound upregulation of the early activation marker CD69 (Table 1 and Fig 1A; CD69 MFI of IL-15 DC-stimulated *vs*. unstimulated NK cells: 219.7±45.9 *vs*. 65.8±23.0, *P*<0.001). This effect was most pronounced in the CD56^bright^ subset, where IL-15 DC stimulation resulted in a >5-fold increase in CD69 MFI compared to basal expression (Fig 1A; *P*<0.001). In line with this, only CD56^bright^ NK cells showed increased expression of CD25 upon IL-15 DC stimulation (Fig 1A; *P*<0.05). The HLA-DR expression level was significantly altered on IL-15 DC-stimulated CD56^dim^ NK cells (Fig 1A, *P*<0.05).

IL-15 DCs and IL-4 DCs differentially affected the expression of NKG2D and the NCRs NKp30 and NKp46. While IL-15 DCs led to a significant increase in the expression of these three markers (Fig 1A; *P*<0.01), IL-4 DCs did not alter the expression of NKG2D and even caused a significant downregulation of NKp30 and NKp46 on the NK cell surface (Fig 1A).

### Except for GM-CSF, NK cell cytokine/chemokine secretion is not affected by IL-15 DCs

**Fig 2:**
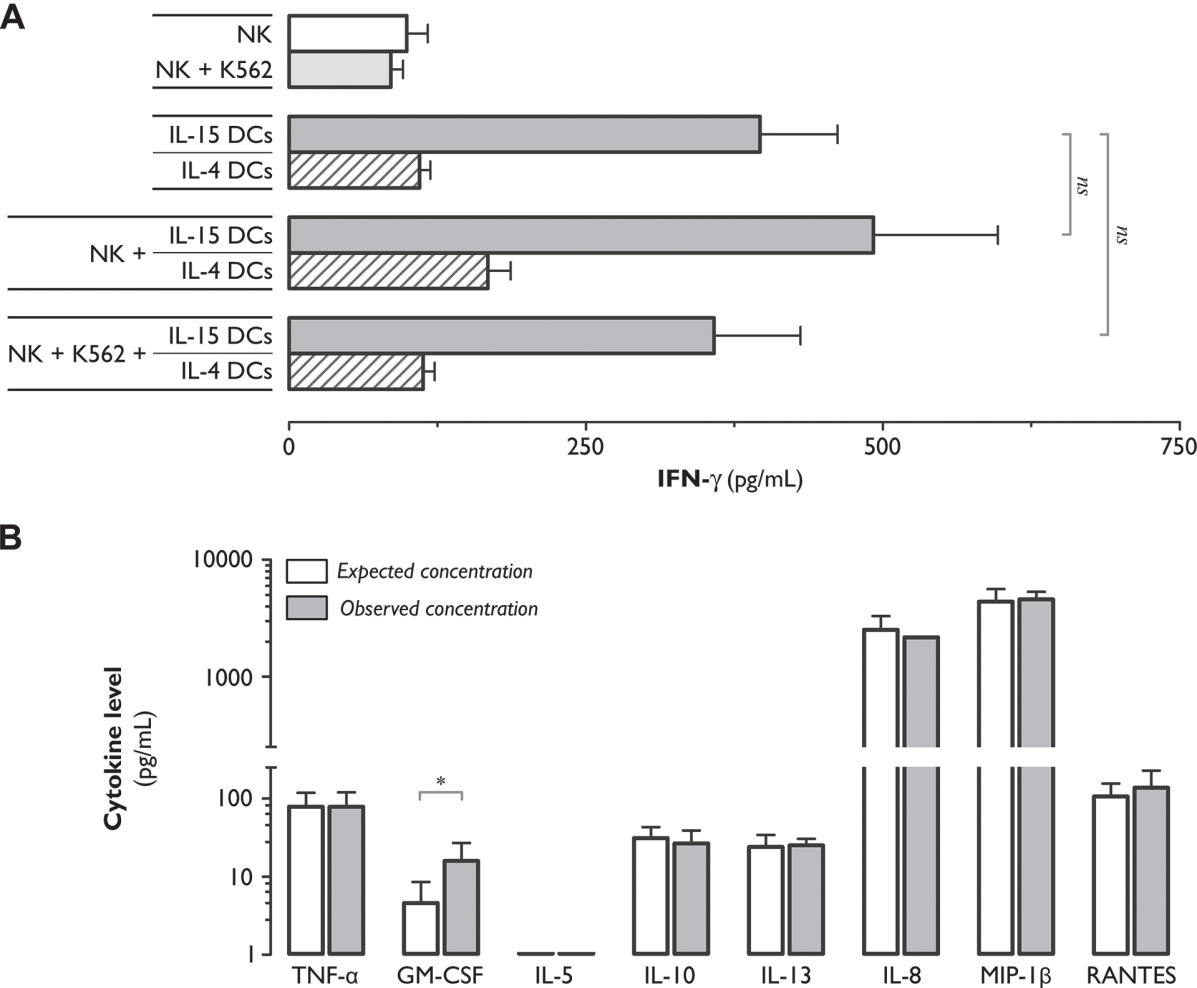
With the exception of GM-CSF, exposure of NK cells to IL-15 DCs does not trigger cytokine or chemokine secretion. (**A**) Bar graphs showing the IFN-γ secretion level (pg/mL), as determined by ELISA, in 48-hr culture supernatants collected from NK cell monocultures, IL-15 DC/IL-4 DC monocultures, and NK+IL-15 DC/IL-4 DC co-cultures. Where indicated, the effect of an additional 4-hr stimulation with the K562 tumor cell line was examined. Values represent means (± SEM) of triplicate cultures from 6 individual donors (ns, not significant). (**B**) Cytokine/chemokine levels (pg/mL; log scale) in 48-hr IL-15 DC-stimulated NK cell culture supernatants, as measured by ECLIA. Observed concentrations (grey bars) were compared with the expected concentrations (white bars) calculated as the sum of the DC and NK cell monoculture concentrations. Values represent means (± SEM) of duplicate measurements of 3 separate donors (*, *P*<0.05; Wilcoxon matched pairs).

The potent phenotypic activation effects of IL-15 DCs on NK cells led us to investigate whether these cells are also capable of modulating the cytokine-secretory function of NK cells. We chose to focus first on IFN-γ, as this is one of the principle cytokines produced by NK cells. As shown in Fig 2A and as anticipated from our previous work [33,35], resting NK cells as well as NK cells exposed to a tumor cell stimulus (e.g. K562) did not secrete relevant amounts of IFN-γ. High concentrations of IFN-γ were detected in supernatants from 48 hr co-cultures of NK cells and IL-15 DCs (492±105 pg/mL), but not IL-4 DCs (Fig 2A). Nevertheless, this level was not significantly different from the level of IFN-γ (397±65 pg/mL) in mono-culture supernatants of IL-15 DCs (Fig 2A; *P*>0.05), which have been shown to be capable of producing IFN-γ themselves [28–30]. We next tested whether additional stimulation of IL-15 DC-exposed NK cells with K562 tumor cells could trigger IFN-γ secretion. As shown in Fig 2A, addition of K562 cells to IL-15 DC-NK cell co-cultures did not result in elevated IFN-γ production (358±73 pg/mL; Fig 2A; *P*>0.05).

We next used a customized ECLIA to determine the secretion of other cytokines/chemokines, including the pro-inflammatory cytokines TNF-α and GM-CSF, the immunoregulatory cytokines IL-5, IL-10 and IL-13, and the chemokines IL-8, MIP-1β and RANTES [36–38]. As shown in Fig 2B, the concentration of GM-CSF in supernatants of IL-15 DC-NK cell co-cultures was found to be >5-fold higher than expected from the mono-cultures. For all other cytokines/chemokines, no changes in secretion levels were observed (Fig 2B; *P*>0.05 for all cytokines listed, except GM-CSF; Wilcoxon matched pairs).

### IL-15 DCs, but not IL-4 DCs, promote NK cell tumoricidal activity

**Fig 3:**
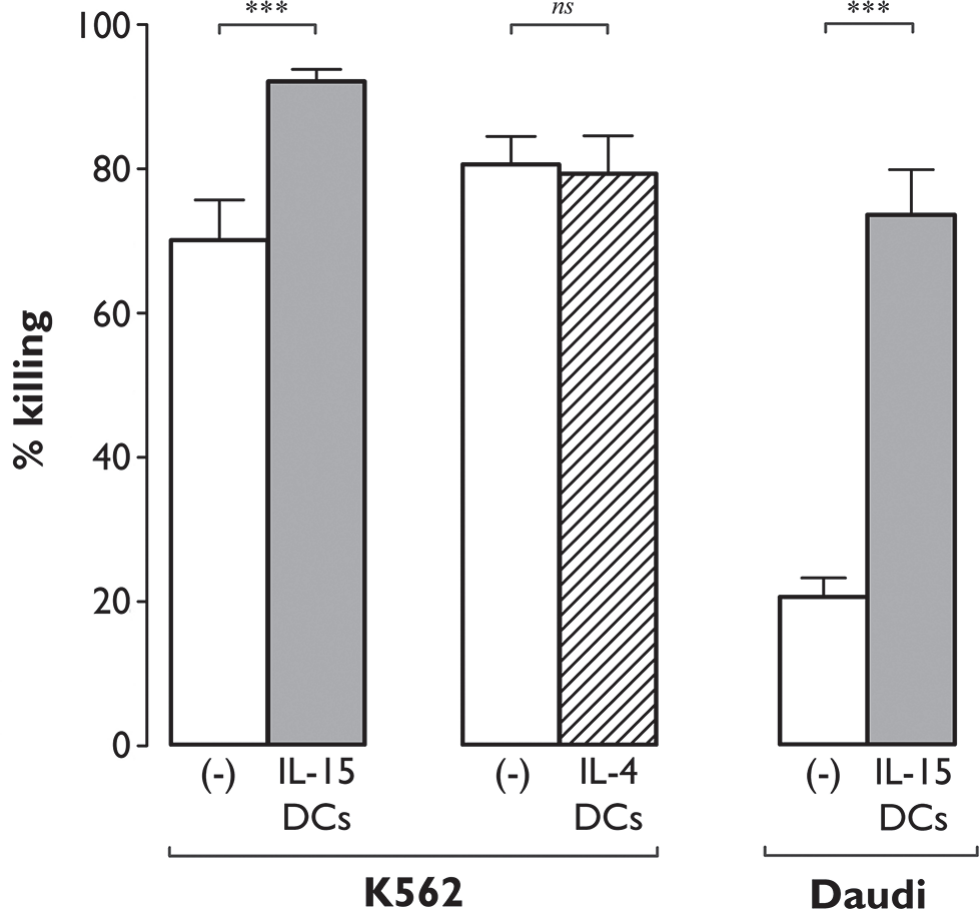
IL-15 DCs but not IL-4 DCs promote NK cell-mediated tumor cell killing. Unstimulated NK cells ((-), white bars) and NK cells exposed for 48 hr to IL-15 DCs (grey bars) or IL-4 DCs (dashed bar) were analyzed by flow cytometry for cytotoxicity against K562 and Daudi tumor cells. Target cell killing was determined by Annexin-V/PI staining after 4 hr incubation at an E:T ratio of 5:1. Results are expressed as mean (± SEM) percentage killing, which was calculated using the formula specified in “Methods”. Data are from two independent experiments involving 10 different donors (***, *P*<0.001; ns, not significant).

To determine whether IL-15 DCs and IL-4 DCs are capable of modulating NK cell cytotoxic function, NK cells were tested for cytotoxicity against various tumor cell targets (NK-sensitive K562 cells and NK-resistant Daudi cells) following a 48-hr stimulation with autologous IL-15 DCs or IL-4 DCs (Fig 3). As shown in Fig 3, resting (unstimulated) NK cells already displayed considerable cytotoxicity towards the K562 tumor cell line, but this activity was nevertheless significantly enhanced by IL-15 DCs (Fig 3; *P*<0.001). IL-4 DCs, on the contrary, did not affect the cytotoxicity of NK cells towards K562 (Fig 3), nor did they induce cytotoxicity against Daudi cells (data not shown). Remarkably, IL-15 DCs caused a 3.6-fold increase in NK cell cytotoxicity towards Daudi (Fig 3; % killing by IL-15 DC-stimulated NK cells *vs*. unstimulated NK cells: 73.6±6.2% *vs*. 20.6±2.6%; *P*<0.001).

### IL-15 DCs harness NK cell cytotoxic function in a contact- and IL-15-dependent manner

**Fig 4:**
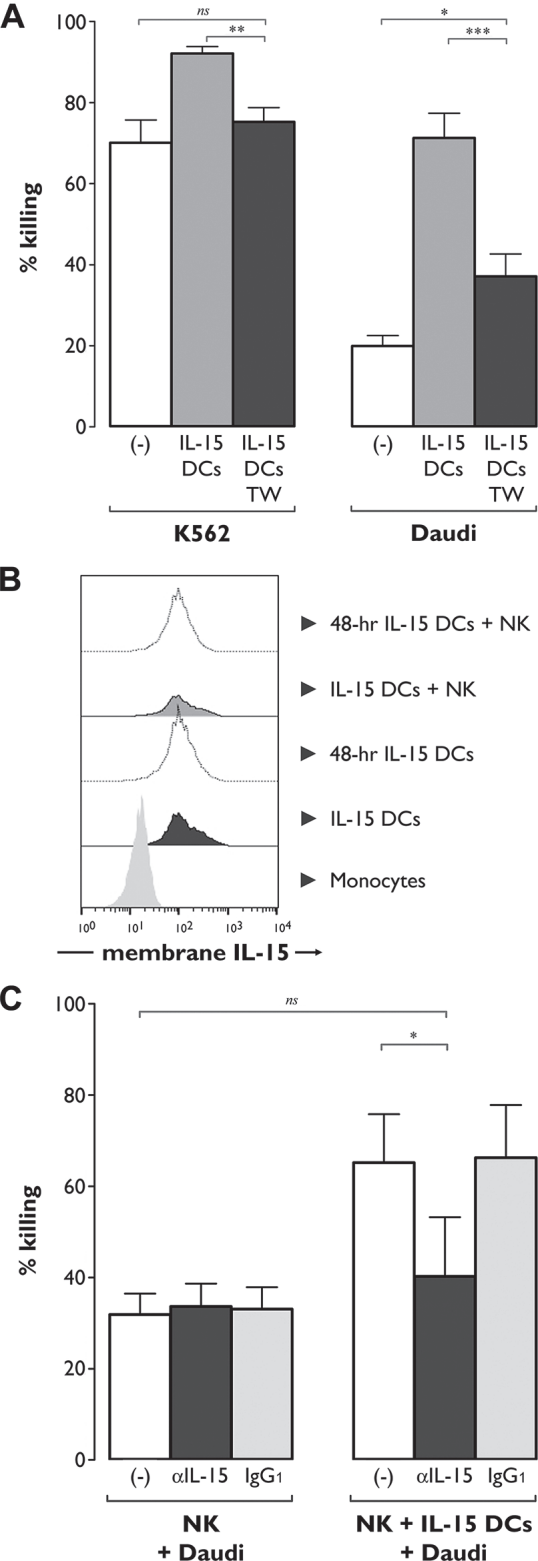
IL-15 DCs enhance NK cell cytotoxicity by a cell contact-dependent mechanism involving membrane-bound IL-15. (**A**) NK cell cytotoxicity was determined after 48-hr co-culture with IL-15 DCs in the absence (grey bars) or presence (TW, dark grey bars) of a Transwell system. NK cells cultured in medium alone for 48 hr ((-), white bars) were used as controls to determine baseline cytotoxicity. K562 and Daudi target cells were added at an E:T ratio of 5:1 and assessed for viability after 4 hr by Annexin-V/PI staining and flow cytometry. Results are expressed as mean (± SEM) percentage killing, which was calculated using the formula specified in “Methods”. Data are from two independent experiments involving 10 different donors (*, *P*<0.05; **, *P*<0.01; ***, *P*<0.001; ns, not significant). (**B**) Flow cytometric analysis of membrane-bound IL-15 expression on monocytes and mature IL-15 DCs at different time points of culture: at harvest of the DCs (IL-15 DCs), at 48 hr post-harvest (48-hr IL-15 DCs), at the start of IL-15 DC-NK cell co-culture (IL-15 DCs + NK) and after 48 hr of co-culture (48-hr IL-15 DCs + NK). One representative sample out of 3 different donors is shown. (**C**) NK cells were cultured in the absence (left panel) or presence of autologous IL-15 DCs (right panel) in medium without neutralizing Ab ((-), white bars) or in medium containing either neutralizing anti-IL-15 mAb (αIL15, dark grey bars) or its corresponding isotype control Ab (IgG_1_, light grey bars). After 16 hr, Daudi tumor cells were added at an E:T ratio of 5:1 for an additional 4 hr after which target cell killing was determined by Annexin-V/PI staining and flow cytometry, as described above. Bars represent mean (± SEM) percentage killing of three individual donors from one experiment (*, *P*<0.05; ns, not significant).

Transwell experiments were performed to investigate whether the observed enhancement of NK cell cytotoxic effector function by IL-15 DCs relied on direct cell-to-cell contact or on soluble factors. As shown in Fig 4A, separation of NK cells and IL-15 DCs by means of a Transwell chamber significantly reduced NK cell cytotoxicity against K562 targets to a level approximating that of unstimulated NK cells (Fig 4A, left panel; *P*<0.01). Lytic activity towards Daudi cells was decreased by 74.5±8.0% by preventing cell contact between NK cells and IL-15 DCs (Fig 4A, right panel; *P*<0.001).

We next examined whether IL-15 itself was involved in this apparently contact-dependent mechanism through which IL-15 DCs harness NK cell cytotoxic function. IL-15 usually does not occur as a soluble factor, but predominantly exists as an IL-15 receptor α (IL-15Rα)-bound form expressed at the DC surface [39–41]. As shown in Fig 4B, IL-15 was indeed found to be expressed at the IL-15 DC surface. Kinetic studies revealed that IL-15 surface expression was maintained for at least 48 hr after DC maturation and also that the expression level of IL-15 was not altered during IL-15 DC-NK cell co-culture (Fig 4B). IL-4 DCs, by contrast, did not express membrane-bound IL-15 (n = 4 of 2 independent experiments, data not shown). Contrary to expectations, we were unable to detect surface expression of IL-15Rα on mature IL-15 DCs (data not shown).

To further clarify the involvement of membrane-bound IL-15, cytotoxicity experiments were repeated, but, prior to co-culture with NK cells, IL-15 DCs were preincubated with anti-IL-15 neutralizing mAbs. Blocking of IL-15 on the IL-15 DC surface reduced the lytic activity of IL-15 DC-stimulated NK cells towards Daudi by 76.3±18.9% (Fig 4C; *P*<0.05), to a level not significantly different from that of unstimulated NK cells (Fig 4C; *P*>0.05).

## Discussion

The contribution of NK cells to the therapeutic efficacy of DC-based cancer immunotherapy is being increasingly recognized in both murine models and human clinical trials [4,5]. Hence, harnessing the NK cell-activating potential of DCs is an important and highly desirable goal towards the design of more effective DC therapies [4]. Given their superior T cell-stimulatory capacity and direct tumoricidal activity, IL-15 DCs have been proposed as an attractive alternative to the “gold-standard” IL-4 DCs currently used for DC-based immunotherapy [22]. Here, for the first time, we performed a head-to-head comparison of the NK cell-stimulatory properties of both DC preparations. The results of the present study clearly show that IL-15 DCs, but not conventional IL-4 DCs, can induce an activated phenotype in NK cells and enhance NK cell cytotoxicity against both NK-sensitive as well as NK-insensitive tumor cells. This augmentation in cytotoxic effector function was found to require cell-to-cell contact and could be almost completely abrogated by blocking membrane-bound IL-15 expressed on the DC surface.

In the first part of this paper, we aimed to compare the phenotypic features of NK cells stimulated with autologous IL-4 DCs *vs*. IL-15 DCs. While some degree of phenotypic activation could be observed after IL-4 DC stimulation, activation was far more pronounced in response to IL-15 DCs. This was evidenced, among other things, by the marked upregulation of CD56 on the NK cell surface. CD56 is generally used as a marker to categorize NK cells into CD56^bright^ and CD56^dim^ cells [7], but it is less well acknowledged that CD56 also serves as a phenotypic activation marker [42–45]. Upon stimulation with IL-15 DCs, both CD56^bright^ and CD56^dim^ subsets were found to upregulate their expression of CD56. A similar observation was made by Bontkes *et al*. [44], showing increased expression of CD56 on NK cells after culture with IL-12 gene-modified DCs. In parallel with CD56, IL-15 DC-stimulated NK cells also exhibited a significant increase of CD69, CD25 and HLA-DR expression, three other known NK cell activation markers [5,46]. The increased expression of HLA-DR is noteworthy, as the frequency of HLA-DR^+^ NK cells has been correlated with clinical responsiveness in DC vaccine-treated acute myeloid leukemia (AML) patients [20]. Besides displaying an activated phenotype, IL-15 DC-stimulated NK cells also showed increased expression of NKG2D and of the NCRs NKp30 and NKp46. IL-4 DCs, by contrast, did not augment the NKG2D expression levels and even downregulated NCR expression. This may be particularly problematic, given the contribution of these molecules to NK cell-mediated tumor control [47,48]. Overall, for all the above-mentioned phenotypic markers, CD56^bright^ NK cells seemed to respond more vigorously to IL-15 DC stimulation as compared to their CD56^dim^ counterparts. This may indicate that IL-15 DCs induce preferential activation of the CD56^bright^ NK cell subset, a notion that was further reinforced by the observed increase in the CD56^bright/dim^ ratio in IL-15 DC-stimulated NK cell cultures. These data corroborate earlier work suggesting that CD56^bright^ NK cells are particularly prone to DC stimulation [8,49,50].

Except for GM-CSF, we observed no significant change in NK cell cytokine/chemokine production in the presence of IL-15 DCs. This was rather unexpected, given that the phenotypic activation changes induced by IL-15 DCs were more pronounced in the CD56^bright^ subset, which is classically considered to be responsible for cytokine production whereas cytotoxicity is typically associated with the CD56^dim^ NK cell subset [7,49]. However, it has become increasingly clear that this functional dichotomy between CD56^bright^ and CD56^dim^ NK cells is not as strict as conventionally postulated [8,37,51–53]. Cytokine and chemokine production is not *per se* a function of the CD56^bright^ subset [37] and several studies have shown that CD56^dim^ NK cells can also function as cytokine producers [37,53]. Within this context and contrary to expectations, Fauriat *et al*. identified CD56^dim^ NK cells as the principal source of cytokines/chemokines when NK cells are stimulated with target cells (e.g. K562 cells) [37]. We were unable to observe any increase in IFN-γ production (Fig 2A) nor other cytokines/chemokines (data not shown) by IL-15 DC-sensitized NK cells that were additionally stimulated with the K562 cell line, thus pointing to an insufficient level of activation of the CD56^dim^ NK cell subsettimulation hemokine productionine.e expected concentrations secretion. s.ted with IL-15 DCs. Therefore, and in view of our phenotypic data reflecting a lower degree of activation of IL-15 DC-stimulated CD56^dim^ NK cells as compared to CD56^bright^ cells, it is possible that the lack of cytokine/chemokine secretion observed in this study may have been due to the relatively weaker ability of IL-15 DCs to stimulate the CD56^dim^ NK cell subset.

While CD56^dim^ NK cells exhibit cytokine responses to target cell stimulation, cytokine/chemokine production by CD56^bright^ NK cells is widely reported to be dependent on the provision of cytokine stimuli [37,54]. Previous work by Cooper *et al*. [54] revealed that stimulation with a single cytokine is usually not sufficient to trigger CD56^bright^ NK cells to release cytokines and/or chemokines. The only exception is GM-CSF, which can be secreted in response to IL-15 without the necessity of co-stimulation with another cytokine [54]. This, together with our finding that IL-15 DCs express IL-15, provides a plausible explanation for the observed increased production of GM-CSF but not of other cytokines/chemokines (which would have required the presence of other cytokines such as IL-12 and/or IL-18 [54]). For example, previous studies have shown that IL-15 alone does not suffice to induce IFN-γ secretion by NK cells, whereas the combination of IL-15 with IL-12 leads to an abundant release of IFN-γ [54,55]. IL-12 is considered to play a primordial role in promoting NK cell IFN-γ secretion [6,38]. Within this context, studies of DC-mediated NK cell activation have shown that the lack or neutralization of DC-derived IL-12 results in the inability of DCs to trigger NK cell IFN-γ secretion [44,50,56,57]. Hence, the fact that no NK cell IFN-γ secretion could be elicited in our experiments may thus be explained by the failure of IL-15 DCs to produce IL-12. Previous work by us and others has characterized IL-15 DCs as poor producers of IL-12, even when a TLR agonist—such as the one in the present study—was used to induced their maturation [25,27,30]. Importantly, the maturation cocktail used in this study included PGE_2_, which is known to compromise the production of IL-12 by DCs and, consequently, their ability to trigger NK cell IFN-γ secretion [58]. However, omitting PGE_2_ from the DC maturation cocktail did not enhance the capacity of IL-15 DCs to stimulate NK cell cytokine/chemokine secretion (data not shown). Thus, PGE_2_ itself cannot be held directly responsible for the weak IL-12-producing capacity of IL-15 DCs and for the failure of NK cells to respond to IL-15 DC stimulation with increased IFN-γ production. In line with this, Harris *et al*. [27] found that TLR-matured IL-15 DCs, despite the absence of PGE_2_ in the maturation cocktail, produce little to no IL-12. These data indicate that the limited capacity of IL-15 DCs to produce IL-12 is intrinsic to these cells and not related to the choice of maturation method or the presence of PGE_2_ in the maturation cocktail.

While no noteworthy changes in cytokine secretion were observed, NK cell cytotoxic function was markedly enhanced by IL-15 DCs. The mechanism behind this enhancement was dependent on IL-15 expressed at the DC surface. The finding that IL-15 DCs were largely ineffective at triggering NK cell cytokine secretion was rather unexpected, in view of earlier studies showing that DCs can harness both the cytotoxic and cytokine-secretory effector functions of NK cells via membrane-bound IL-15 [39,40,43,55,59,60]. However, in these studies, triggering of NK cell cytokine secretion required IL-15 to be presented “in trans” to the NK cells via IL-15Rα [40,55]. We were unable to detect any substantial membrane expression of IL-15Rα on IL-15 DCs, adding another possible explanation for their apparent inability to stimulate the cytokine-secretory function of NK cells. The lack of IL-15Rα expression on IL-15 DCs may be related to their similarity with Langerhans cells [22,23,25], which are unable to upregulate IL-15Rα [43]. Furthermore, the discordant expression of IL-15 and IL15Rα should not be entirely unexpected, since monocytes and DCs can also express IL-15 as a transmembrane protein independently of IL-15Rα [27,41,61,62].

Although the lytic activity of NK cells is generally attributed to the CD56^dim^ subset, our data suggest a role for the CD56^bright^ subset in the observed enhancement of NK cell cytotoxicity following IL-15 DC stimulation. CD56^bright^ NK cells, when activated with cytokines such as IL-15, can become cytotoxic [51,52,63] and even mediate lysis of target cells that are usually regarded as “NK-resistant” [51], such as the Daudi tumor cell line [64]. In view of the fact that a similar lytic profile was observed in this study and the above-mentioned observation that IL-15 DCs mediate a more robust activation of the CD56^bright^ NK cell subset, it is conceivable that CD56^bright^ NK cells are involved in the enhanced NK cell cytotoxicity after IL-15 DC stimulation. Furthermore, IL-15 DCs induced several phenotypic changes in CD56^bright^ NK cells that are compatible with enhanced lytic potential, such as increased expression of CD56, CD69, HLA-DR, NKG2D, NKp30 and NKp46. For all these phenotypic markers, a direct relation between their expression and cytotoxic activity has been established [19,42,44–46,65,66], further supporting our contention that the CD56^bright^ NK cell subset significantly contributed to the cytotoxicity of IL-15 DC-activated NK cells. The ability of IL-15 DCs to specifically harness the CD56^bright^ NK cell compartment may be of particular relevance for immunotherapy since CD56^bright^ NK cells have been identified as the predominant tumor-infiltrating NK cell population [8,67]. In line with this, recent studies have assigned a key role to CD56^bright^ NK cells in the cytotoxic effector response against various malignancies, including AML [63] and melanoma [68].

### Conclusions

In summary, this study offers additional insights into the mechanisms whereby DCs regulate NK cell functions and reveals that human DCs can harness the cytotoxic effector function of NK cells via membrane-bound IL-15 even in the apparent absence of IL-15Rα. Such membrane-bound IL-15 is not sufficient to trigger NK cell IFN-γ secretion, which likely requires coordinate expression of IL-15Rα [40,55] or secretion of soluble cytokines (most notably IL-12) [5,38]. We found that IL-15 DCs are armed with membrane-bound IL-15 and thereby equipped with the capacity to potentiate NK cell cytotoxicity, whereas “gold-standard” IL-4 DCs used in clinical studies are not. This finding has direct translational implications, in view of the increasingly appreciated contribution of NK cells to the therapeutic activity of DC-based immunotherapies.

## Supporting Information

S1 Fig IL-15 DCs start to induce phenotypic NK cell activation already after 24 hr of co-culture. (**A**) Bar graphs showing the MFI (± SEM) of CD56, CD69, CD25, HLA-DR, NKG2D, NKp30 and NKp46 on unstimulated NK cells ((-), white bars) and NK cells stimulated for 24 hr with autologous IL-15 DCs (grey bars) or IL-4 DCs (dashed bars). Expression levels of the indicated markers are shown for the total CD56^+^ NK cell population, as well as for the CD56^bright^ and CD56^dim^ NK cell subpopulations. Data are from one experiment with 6 different donors (*, *P*<0.05; **, *P*< 0.01; ***, *P*<0.001; ns, not significant). (**B**) Bar graphs showing the relative distribution of CD56^bright^ and CD56^dim^ subsets in unstimulated NK cells ((-)) and in NK cells cultured for 24 hr with IL-15 DCs or IL-4 DCs. Data are expressed as mean (± SEM) percentages of 6 donors from 1 experiment (ns, not significant).
(TIF)

S1 Table Percentage increase in cell surface marker positivity compared with baseline after 24 hr stimulation of NK cells with either IL-15 DCs or IL-4 DCs. Pairwise comparison of the expression of CD56, CD69, CD25, HLA-DR, NKG2D, NKp30 and NKp46 on NK cells (total CD56^+^ NK cell population, CD56^bright^ subset and CD56^dim^ subset) after 24-hr incubation with IL-15 DCs or IL-4 DCs. Results are presented as mean (± SD) percentage increase in positivity of the indicated cell surface marker as compared with the baseline expression on unstimulated NK cells (calculated using the Overton histogram subtraction algorithm by subtracting the unstimulated NK cell control histogram from the DC-stimulated NK cell sample histogram). Asterisks indicate a statistically significant difference in cell surface marker expression between IL 15 DC-stimulated and IL-4 DC-stimulated NK cells (n = 6; *, *P*<0.05).
(DOC)
