# Supplementary figures and images for: Interleukin-15 Dendritic Cells Harness NK Cell Cytotoxic Effector Function in a Contact- and IL-15-Dependent Manner
Source: PLoS One. 2015 May 7;10(5):e0123340. doi: 10.1371/journal.pone.0123340 (PMC4423923; doi:10.1371/journal.pone.0123340)

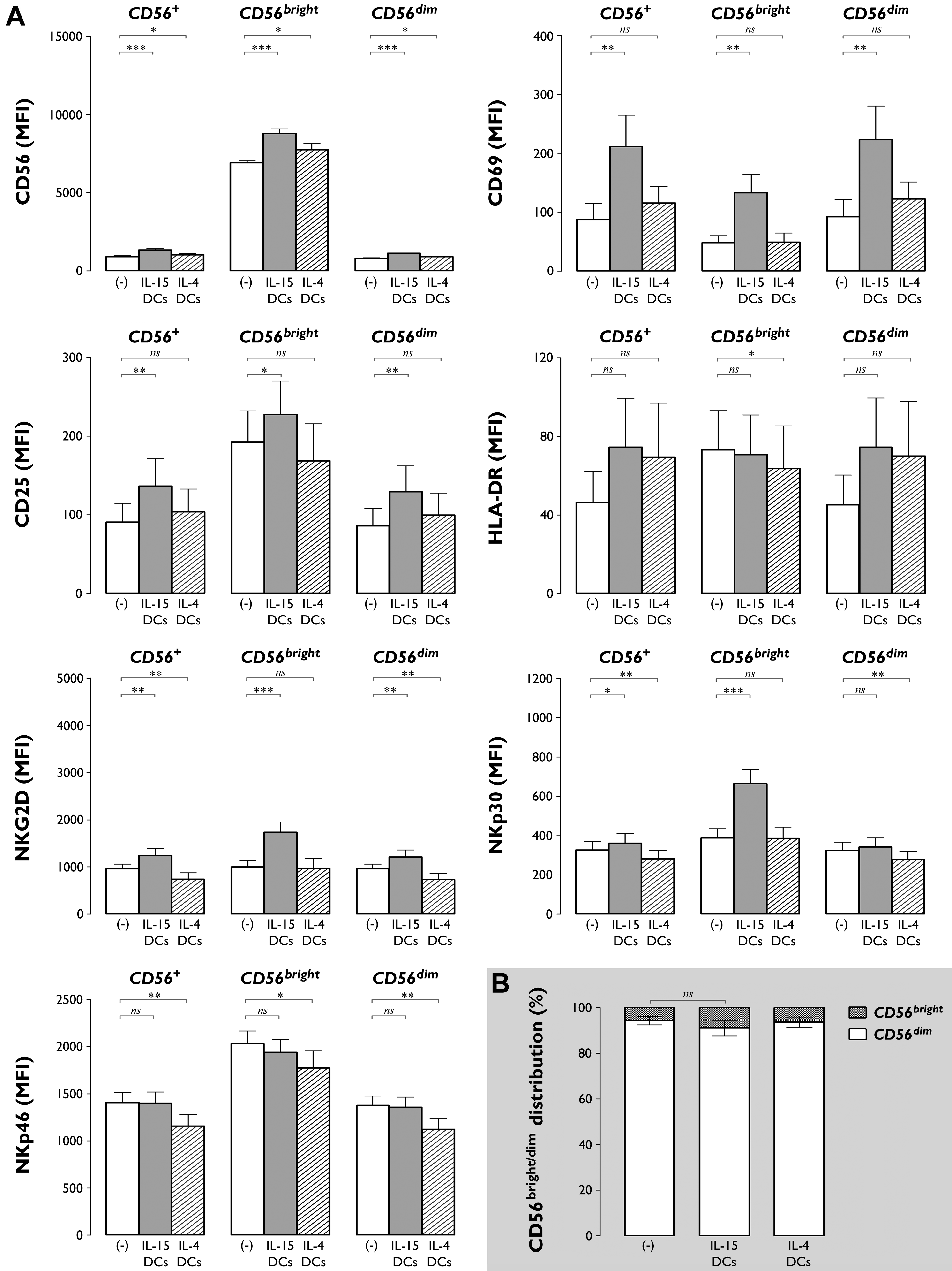

Supplement: S1 Fig — (A) Bar graphs showing the MFI (± SEM) of CD56, CD69, CD25, HLA-DR, NKG2D, NKp30 and NKp46 on unstimulated NK cells ((-), white bars) and NK cells stimulated for 24 hr with autologous IL-15 DCs (grey bars) or IL-4 DCs (dashed bars). Expression levels of the indicated markers are shown for the total CD56+ NK cell population, as well as for the CD56bright and CD56dim NK cell subpopulations. Data are from one experiment with 6 different donors (*, P<0.05; **, P< 0.01; ***, P<0.001; ns, not significant). (B) Bar graphs showing the relative distribution of CD56bright and CD56dim subsets in unstimulated NK cells ((-)) and in NK cells cultured for 24 hr with IL-15 DCs or IL-4 DCs. Data are expressed as mean (± SEM) percentages of 6 donors from 1 experiment (ns, not significant). (TIF) [file pone.0123340.s001.tif]
