# Supplementary material for: Interleukin-15 Dendritic Cells Harness NK Cell Cytotoxic Effector Function in a Contact- and IL-15-Dependent Manner
Source: PLoS One. 2015 May 7;10(5):e0123340. doi: 10.1371/journal.pone.0123340 (PMC4423923; doi:10.1371/journal.pone.0123340)
Supplement: S1 Table — Pairwise comparison of the expression of CD56, CD69, CD25, HLA-DR, NKG2D, NKp30 and NKp46 on NK cells (total CD56+ NK cell population, CD56bright subset and CD56dim subset) after 24-hr incubation with IL-15 DCs or IL-4 DCs. Results are presented as mean (± SD) percentage increase in positivity of the indicated cell surface marker as compared with the baseline expression on unstimulated NK cells (calculated using the Overton histogram subtraction algorithm by subtracting the unstimulated NK cell control histogram from the DC-stimulated NK cell sample histogram). Asterisks indicate a statistically significant difference in cell surface marker expression between IL 15 DC-stimulated and IL-4 DC-stimulated NK cells (n = 6; *, P<0.05). (DOC) [file pone.0123340.s002.doc]

Table S1. Percentage increase in cell surface marker positivity compared with baseline after 24 hr stimulation of NK cells with either IL-15 DCs or IL-4 DCs

|  | **CD56^+^ NK cells** | | **CD56^bright^ NK cells** | | **CD56^dim^ NK cells** | |
| --- | --- | --- | --- | --- | --- | --- |
|  | ***IL-15 DCs*** | ***IL-4 DCs*** | ***IL-15 DCs*** | ***IL-4 DCs*** | ***IL-15 DCs*** | ***IL-4 DCs*** |
| **CD56** | 25.1±2.9% | 10.3±3.6%* | 30.3±10.4% | 18.7±16.0%* | 25.0±3.9% | 10.4±4.4%* |
| **CD69** | 24.8±6.6% | 8.1±6.8%* | 27.2±7.2% | 5.9±2.8%* | 25.5±6.0% | 8.4±7.0%* |
| **CD25** | 13.8±2.8% | 4.0±2.2% | 13.2±8.5% | 0.0±8.3% | 13.4±3.6% | 4.4±2.1% |
| **HLA-DR** | 10.0±4.1% | 5.5±4.6%* | 2.8±1.1% | 1.7±1.9% | 10.3±4.3% | 5.8±4.6%* |
| **NKG2D** | 16.4±7.1% | 2.4±5.6%* | 29.8±8.6% | 6.3±4.2%* | 15.3±7.8% | 2.4±5.8%* |
| **NKp30** | 5.9±3.6% | 1.8±3.1% | 31.9±9.6% | 5.0±4.8% | 3.9±3.8% | 1.7±3.1% |
| **NKp46** | 3.7±2.8% | 0.2±0.4%* | 3.0±3.2% | 1.7±2.2% | 3.6±2.9% | 0.2±0.3%* |

Pairwise comparison of the expression of CD56, CD69, CD25, HLA-DR, NKG2D, NKp30 and NKp46 on NK cells (total CD56^+^ NK cell population, CD56^bright^ subset and CD56^dim^ subset) after 24-hr incubation with IL-15 DCs or IL-4 DCs. Results are presented as mean (± SD) percentage increase in positivity of the indicated cell surface marker as compared with the baseline expression on unstimulated NK cells (calculated using the Overton histogram subtraction algorithm by subtracting the unstimulated NK cell control histogram from the DC-stimulated NK cell sample histogram). Asterisks indicate a statistically significant difference in cell surface marker expression between IL 15 DC-stimulated and IL-4 DC-stimulated NK cells (n = 6; *, *P*<0.05).
